# Prognostic relevance of caspase 8 -652 6N InsDel and Asp302His polymorphisms for breast cancer

**DOI:** 10.1186/s12885-016-2662-x

**Published:** 2016-08-09

**Authors:** J. D. Kuhlmann, A. Bankfalvi, K. W. Schmid, R. Callies, R. Kimmig, P. Wimberger, W. Siffert, H. S. Bachmann

**Affiliations:** 1 Department of Gynecology and Obstetrics, Medical Faculty and University Hospital Carl Gustav Carus, Technische Universität Dresden, Dresden, Germany; 2 German Cancer Consortium (DKTK), Dresden and German Cancer Research Center (DKFZ), Heidelberg, Germany; 3 Institute of Pathology and Neuropathology, University Hospital Essen, University of Duisburg-Essen, Essen, Germany; 4 Department of Gynecology and Obstetrics, West German Cancer Center, University of Duisburg-Essen, Essen, Germany; 5 Institute of Pharmacogenetics, University Hospital Essen, University of Duisburg-Essen, Hufelandstr. 55, 45147 Essen, Germany; 6 National Center for Tumor Diseases, Partner Site Dresden, Dresden, Germany

**Keywords:** Caspase 8, Polymorphism, *CASP8* -652 InsDel, *CASP8* Asp302His, Breast cancer, Prognostic biomarker

## Abstract

**Background:**

The minor allele of two caspase 8 polymorphisms, namely *CASP8* -652 6N InsDel (rs3834129) and *CASP8* Asp302His (rs1045485), were repeatedly associated with reduced breast cancer susceptibility. Contrarily, the presence of the -652 6N Del or the *CASP8* 302His variant was reported to be an unfavorable prognostic factor in colorectal cancer or neuroblastoma. However, prognostic relevance of these genetic variants for breast cancer is completely unknown and is therefore adressed by the current study.

**Methods:**

Genotyping was performed by pyrosequencing. Caspase 8 mRNA expression was quantified by comparative RT-qPCR.

**Results:**

We observed an allele-dose dependent association between *CASP8* -652 6N InsDel and caspase 8 mRNA expression in breast cancer tissue, with homozygous deletion carriers showing lowest relative caspase 8 expression (*p* = 0.0131). Intriguingly, the presence of the -652 6N Del or the 302His variant was shown to be a negative prognostic factor for breast cancer in terms of an allele-dose dependent influence on overall survival (OS, *p* = 0.0018, *p* = 0.0150, respectively). Moreover, both polymorphisms were independent predictors of OS after adjusting for co-variats (*p* = 0.007, *p* = 0.037, respectively). Prognostic relevance of both polymorphisms were confirmed to be independent from each other and combined analysis of diplotypes revealed an additive influence upon OS (*p* = 0.0002).

**Conclusion:**

This is the first report, showing negative and independent prognostic impact of the *CASP8* -652 6N Del and the 302His variant for breast cancer. Our data provide rationale to further validate clinical utility of these polymorphisms for breast cancer and to extend this investigation to a broad scope of other malignancies.

**Electronic supplementary material:**

The online version of this article (doi:10.1186/s12885-016-2662-x) contains supplementary material, which is available to authorized users.

## Background

Programmed cell death, also referred to as apoptosis, physiologically occurs in multicellular organisms and its aberration has important implications in cancer biology. Among the death receptor signaling pathway, the initiator Caspase 8, a 55 kDa cysteine protease, plays an important role in intrinsic and extrinsic apoptosis induction. In terms of the intrinsic apoptosis pathway, caspase-8 activates the death inducing signaling complex (DISC), which in turn induces downstream effector caspase-3, finally resulting in apoptosis [1, 2]. Among the extrinsic pathway, caspase-8 cleaves the Bcl-2 related protein Bid, which in turn induces cytochrome c release from mitochondria and caspase-3 activation, likewise resulting in apoptosis [1, 2].

**Fig. 1:**
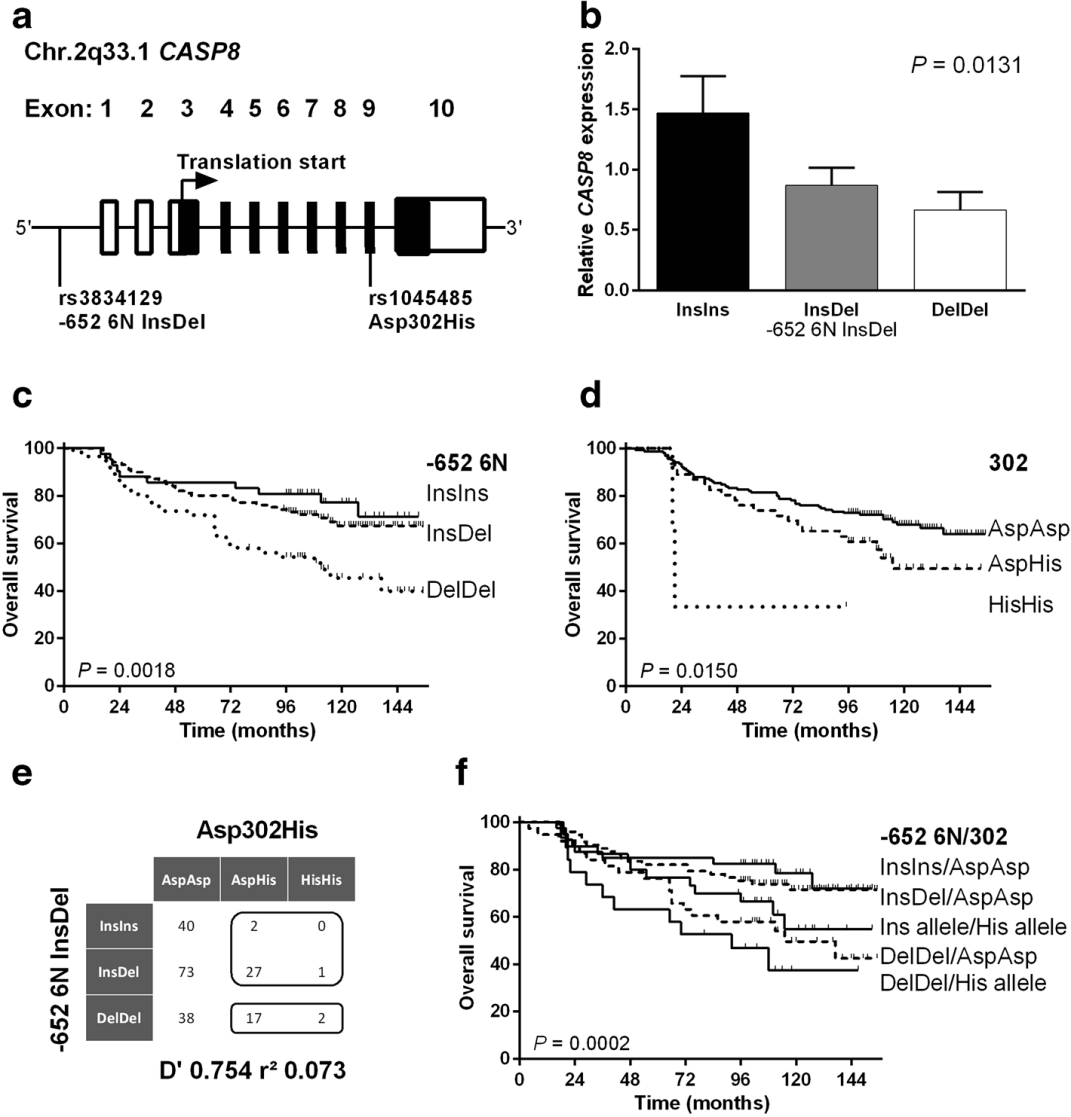
Analysis of caspase-8 polymorphisms in breast cancer patients. **a** Schematic overview of the caspase-8 gene and localization of the caspase 8 polymorphisms of interest. The non-coding *CASP8* -652 6N InsDel polymorphism, a functional 6 bp deletion, is located in the promoter region of the *CASP8* gene, whereas the Asp302His polymorphism is located in the coding region (exon 9). **b** *CASP8* mRNA expression in primary breast cancer tissue by the -652 6N InsDel genotypes. Indicated p-value was calculated by linear ANOVA. **c**–**d** Kaplan-Meier curves comparing overall survival of breast cancer patients by the -652 6N InsDel or *CASP8* Asp302His genotypes, respectively. **e** Overview of possible diplotypes. Five patients belonged to rare diplotypes and needed to be analyzed together with other patients. The figure shows how we joined these rare diplotype carriers with the common ones. **f** Kaplan-Meier curves comparing overall survival of breast cancer patients with regard to the diplotypes of the -652 6N InsDel and Asp302His polymorphisms

Nearly at the same time, two *CASP8* polymorphisms, namely *CASP8* -652 AGTAAG InsDel (-652 6N Del, rs3834129) and *CASP8* Asp302His (rs1045485) were described in key publications [3, 4]. The non-coding *CASP8* -652 6N InsDel polymorphism, a functional 6-bp deletion located in the promoter region of the *CASP8* gene, has been associated with reduced *CASP8* mRNA expression and concomitantly impaired caspase-8 activity and reduced “activation induced cell death” (AICD) in stimulated T-lymphocytes [3]. The second polymorphism, *CASP8* Asp302His, is located in the coding region of caspase 8 and results in aspartic acid to histidine substitution (Fig. 1a). Although instructive data on the functionality of this polymorphism are missing, it was hypothesized that the Asp302His change could likewise impair caspase-8 function, possibly by negatively affecting its auto processing or its catalytic activity [5].

Given that the above mentioned caspase-8 variants were suggested to impair caspase-8 function and to interfere with cell death of T-lymphocytes, which has essential consequences on immune surveillance of malignancies, a variety of studies on different cancer entities investigated, whether these polymorphisms may influence cancer susceptibility. These studies, also including meta-analyses, were primarily performed on breast cancer, but also on other cancer entities, such as colorectal-, ovarian- or gastric cancer. The majority of investigators observed an association of the *CASP8* -652 6N Del or the *CASP8* Asp302His variant with reduced cancer susceptibility [3, 5–12]. However these insights were not entirely conclusive, since some other studies failed to confirm a significant cancer protective effect of these caspase 8 variants [13–17]. Furthermore, since they are in linkage disequilibrium, it remains unclear, whether both or only one of these polymorphisms has an impact on cancer risk.

Beside the extensively studied role of the above mentioned caspase 8 polymorphisms in cancer susceptibility, recent approaches also started to investigate, whether these polymorphisms also influence the outcome of cancer in patients with already existing disease. In this context, recent pilot investigations reported a negative prognostic impact for the *CASP8* -652 Del allele or the *CASP8* 302His allele for patients with colon cancer or neuroblastoma, respectively [18, 19]. However, although breast cancer was primarily adressed by recent *CASP8* -652 6N Del and *CASP8* Asp302His susceptibility studies, surprisingly, prognostic relevance of these caspase 8 polymorphisms has not been investigated in breast cancer so far.

Hypothesizing that a functional polymorphism, which is involved in cancer susceptibility, is also likely to influence the outcome of a given cancer, we took advantage of a historic breast cancer cohort of clinically documented primary breast cancer patients and investigated prognostic significance of *CASP8* -652 6N Ins/Del and *CASP8* Asp302His in terms of an exploratory analysis.

## Methods

### Patient characteristics

The present study refers to a clinically documented historic breast cancer cohort, being recruited between 1989 and 1993 at the Department of Gynecology and Obstetrics, University Hospital of Essen, Germany [20]. In this context, a total of 200 consecutive Caucasian patients of German ancestry, who were diagnosed and operated for histologically confirmed primary breast cancer, were enrolled into this study. This study was approved by the ethics committee of the University Hospital of Essen, Germany (06-3126) and was performed, according to the Declaration of Helsinki. Since this study was performed retrospectively on a historic breast cancer cohort, no patient’s consent was required. Characteristics for primary breast cancer patients are summarized in Table 1. The majority of patients had small tumors and 57.5 % were node-negative. Most patients had invasive ductal breast cancer (68 %) and moderately or poorly differentiated tumors were predominant (63 %). Survival data of these patients were obtained from the patients’ files or the local municipal registry.

**Table 1:** Clinico-pathological characteristics at primary diagnosis and -652 6N InsDel genotype distribution

	All	*CASP8* -652 6N InsDel genotype			*P*-value
		InsIns	InsDel	DelDel	
n (%)	200	42 (21.0)	101 (50.5)	57 (28.5)	
Age at diagnosis (years ± SD)	56.46 ± 12.1	55.00 ± 10.4	57.19 ± 12.4	56.26 ± 12.6	0.676
Tumor type					
Ductal	136 (68.0)	28 (20.6)	68 (50.0)	40 (29.4)	0.243
Lobular	43 (21.5)	12 (27.9)	23 (53.5)	8 (18.6)	
Others	21 (10.5)	2 (9.5)	10 (47.6)	9 (42.9)	
Tumor size (mm ± SD)	24.20 ± 16.8	23.51 ± 19.2	25.11 ± 17.1	23.07 ± 14.5	0.837
Tumor stage					
pT_1_	107 (53.5)	24 (22.4)	53 (49.5)	30 (28.1)	0.540
pT_2_	71 (35.3)	11 (15.5)	39 (54.9)	21 (29.6)	
pT_3+4_	22 (11.0)	7 (31.8)	9 (40.9)	6 (27.3)	
Lymph node status					
pN_0_	115 (57.5)	24 (20.9)	61 (53.0)	30 (26.1)	0.592
pN_+_	85 (42.5)	18 (21.2)	40 (47.1)	27 (31.8)	
UICC stage					
I	76 (38.0)	17 (22.4)	37 (48.7)	22 (28.9)	0.442
II	78 (39.0)	13 (16.7)	46 (59.0)	19 (24.4)	
III + IV	46 (23.0)	12 (26.0)	18 (39.1)	11 (23.9)	
Grade					
1	71 (37.4)	16 (22.5)	35 (49.3)	20 (28.2)	0.379
2	68 (35.8)	10 (14.7)	40 (58.8)	18 (26.5)	
3	51 (26.8)	13 (25.5)	21 (41.2)	17 (33.3)	
Estrogen receptor status					
negative	53 (32.5)	12 (7.4)	29 (17.8)	12 (7.4)	
positive	110 (67.5)	25 (15.3)	50 (30.7)	35 (21.5)	0.432
Her2 status					
negative	138 (83.6)	30 (18.1)	68 (41.2)	40 (24.2)	0.835
positive	27 (16.4)	5 (3.0)	15 (9.1)	7 (4.2)	
Treatment					
Surgical treatment					
breast conserving	50 (25.0)	10 (20.0)	23 (46.0)	17 (34.0)	0.604
ablative	150 (75.0)	32 (21.3)	78 (52.0)	40 (26.7)	
Adjuvant therapy					
no adjuvant therapy	112 (56.0)	22 (19.6)	62 (57.1)	28 (25.0)	0.285
Tam and/or CMF	88 (44.0)	20 (22.7)	39 (44.3)	29 (33.0)	

*Tam* Tamoxifen, *CMF* cyclophosphamide, methotrexate and 5 fluorouracil. Data are numbers with percentages given in brackets. Categorical variables were analyzed by χ^2^ statistics. *P* values were calculated using ANOVA for continuous variables

### DNA extraction and caspase 8 genotyping

Genomic DNA was isolated as previously described [20]. Briefly, several 10–20 μm thick sections from routinely processed paraffin blocks (non-tumorous breast or lymph node specimens) were dewaxed in xylene, washed in ethanol and centrifuged. The supernatant was removed and the open microfuge tube was incubated at 45 °C until the ethanol had evaporated. DNA was purified with the QIAamp DNA Mini Kit (Qiagen, Hilden, Germany). The tissue pellet was re-suspended in 180 μl of buffer ATL/20 μl proteinase K and incubated overnight at 56 °C. Further processing of the samples was done according to the manufacturer’s instructions. *CASP8* -652 6N InsDel and *CASP8* Asp203His genotypes were determined by pyrosequencing (Biotage, Uppsala, Sweden), according to the manufacturer’s instruction. First, the genomic caspase 8 regions of interest were amplified using the “slowdown” polymerase chain reaction (PCR) [21], with the following primer sequences: -652 6N forward: 5′ BIOTIN-AACTTGCCCAAGGTCACG 3′, -652 6N reverse: 5′ TGAGGTCCCCGCTGTTAA 3′, 302 forward: 5′ GACCACGACCTTTGAAGAGCT 3′, and 302 reverse: 5′ BIOTIN-AGATTTGCTCTACTGTGCAGTCA 3′. PCR products were analyzed by pyrosequencing using sequencing primers -652 6N 5′ GTAATTCTTGCTCTGCC 3′ and 302 5′ TGAGATCAAGCCCCA 3′ on the PSQ96 system, according to the manufacturer’s instructions (Biotage, Uppsala, Sweden). Results were analyzed using the proprietary PSQ96 SNP software. Re-genotyping of 30 randomly selected samples to control for genotype failures revealed 100 % concordance with the previously obtained results.

### RNA extraction and Quantitative Real-Time PCR

Total RNA was extracted from snap-frozen breast cancer tissue with the Qiagen RNeasy kit and according to the manufacturer’s instructions. One μg of total RNA was applied for cDNA synthesis with oligo dT primers (Roche, Mannheim, Germany) and Superscript II reverse transcriptase (Invitrogen, Karlsruhe, Germany). Relative *CASP8* mRNA expression was evaluated by RT-qPCR analysis, using the SYBR Green PCR kit (Qiagen, Hilden, Germany), according to the manufacturer’s instructions. Quantitative RT-qPCR was performed using the ABI-7500 system (Applied Biosystems, Darmstadt, Germany). Primer sequences were designed, in order to detect all caspase 8 isoforms (forward: 5′ AAA TCT CCA AAT GCA AAC T 3′, reverse: 5′ATC TTC AGC AGG CTC TTG T 3′). Data were analyzed using the ABI Sequence Detection software (version 1.2.3). The Cq-threshold was adjusted to a fluorescent level above the background signal and within the linear range of each amplification plot. Melting curves were drawn after each PCR run in order to ensure that a single and specific PCR-product was generated. All samples, including non-RT (without reverse transcriptase) and no-template controls were assayed in triplicates. Mean Cq-values and deviations between the triplicates were calculated. Samples with a Cq deviation >0.5 or with any evidence for melting curve abnormality were repeated. Caspase 8 expression values were normalized to human ß-actin expression as housekeeping reference [22]. Reported normalized relative expression values were calculated by the 2^-**deltaCq**^ method and corresponded to 2 ^**-[Cq(caspase 8) - Cq(β-actin)]**^.

### Statistical analysis

Statistical analyses were performed using GraphPad Prism 6.0 (GraphPad Software, LaJolla, CA, USA) and SPSS software version 21.0 (IBM, Armonk, NY, USA). Clinical variables and genotypes were compared using either Student’s t test, ANOVA for continuous variables or Pearson’s Chi^2^ test for categorical data. Control for deviation from the Hardy–Weinberg equilibrium was conducted using a publically available Hardy–Weinberg equilibrium calculator [23]. Linkage disequilibrium and haplotypes were assessed using Haploview [24]. Kaplan-Meier plots and the log-rank test for trend were used to retrospectively evaluate the relationship between *CASP8* Asp302His genotypes, *CASP8* -652 6N InsDel genotypes, *CASP8* diplotypes, and outcome between the date of primary diagnosis and the end of follow-up. Both univariate analysis and stepwise backward multivariable Cox regression analysis were used to analyze the effect of genotypes and diplotypes of the *CASP8* polymorphisms on clinical outcome. Hazard ratios (HR) and 95 % confidence intervals (95 % CI) were calculated based on the Cox regression model. Differences with *p*-values <0.05 were considered significant; all p-values are two-tailed.

## Results

### *CASP8* -652 6N InsDel polymorphism influences *CASP8* mRNA expression in malignant breast cancer tissue in an allele-dose specific manner

The *CASP8* -652 6N InsDel polymorphism was previously shown to influence caspase 8 mRNA expression in lymphocytes in an allele-dose specific manner [3]. To interrogate, whether this effect may also apply to malignant breast cancer tissue, we quantified Caspase 8 mRNA expression in 55 breast cancer patients from which snap-frozen cancer tissue for RNA-extraction was available. Normalized expression data were used to test if there are mean differences in caspase 8 expression by genotype groups. Interestingly, cancer tissues being homozygous for the deletion (*n* = 15), displayed lowest relative caspase 8 mRNA expression, followed by heterozygous samples (*n* = 23). Highest expression levels were found in tissues bearing the homozygous insertion variant (*n* = 17) (*p* = 0.013, Fig. 1b).

Conclusively, we observed a significant allele-dose dependent association between *CASP8* -652 6N Del allele and decreased caspase 8 mRNA expression in primary breast cancer tissue.

### Prognostic relevance of *CASP8* -652 6N InsDel for breast cancer

In 57/200 patients (28.5 %), homozygosity for the deletion variant (DelDel) was observed, whereas 101/200 patients (50.5 %) were heterozygous (InsDel) and 42/200 patients (21.0 %) showed an InsIns genotype (Table 1). No significant deviation from Hardy-Weinberg equilibrium was detectable (*p* = 0.824) and the observed genotype distribution as well as the allelic frequencies (f_Ins_ = 0.463) were comparable to those previously reported in cancer cases and healthy controls of European ancestry [13, 25].

Subsequently, we investigated, whether *CASP8* -652 6N InsDel genotyping may provide prognostically relevant information for breast cancer patients. After confirming that clinico-pathological characteristics consistently lacked significant associations with the underlying genotypes (Table 1), Kaplan-Meier analysis was performed, in order to determine prognostic relevance of the *CASP8* -652 6N InsDel polymorphism. Intriguingly, an allele-dose dependent influence of *CASP8* -652 6N InsDel upon OS was observed (Fig. 1c, *p* = 0.0018), with homozygous deletion carriers at highest risk of death (hazard ratio (HR) = 2.384; 95 % confidence interval (CI) = 1.31–5.48; *p* = 0.007; Table 2). Moreover, multivariable Cox-regression analysis revealed the *CASP8* -652 6N DelDel genotype to be an independent prognostic factor for reduced OS (HR = 2.769; 95 % CI = 1.32–5.81; *p* = 0.007; Table 2).

**Table 2:** Risk of death by uni- and multivariable -652 6N InsDel Cox-regression analyses

Variable	Hazard Ratio	95 % CI	*P*
*Univariate Analysis*			
-652 6N del			
InsIns	1^a^		
InsDel	1.316	0.65–2.68	0.450
DelDel	2.384	1.31–5.48	0.007
*Multivariable Analysis*			
-652 6N del			
InsIns	1^a^		
InsDel	1.490	0.72–3.10	0.286
DelDel	2.769	1.32–5.81	0.007
Age (per year)	1.002	0.98–1.02	0.863
Tumor type			
ductal	1^a^		
lobular	1.909	1.00–3.64	0.050
others	1.713	0.88–3.35	0.115
Tumor stage			
T_1_	1^a^		
T_2-4_	1.798	1.09–2.98	0.023
Nodal status			
negative	1^a^		
positive	3.681	2.13–6.35	<0.001
Grade			
1	1^a^		
2	1.044	0.56–1.94	0.892
3	1.776	0.89–3.53	0.102

^a^Reference group

In our historic breast cancer cohort, routine assessment of ER and Her2 receptor status had not yet been diagnostic standard. Nevertheless, ER and Her2 receptor data were available in 163/200 and 165/200 cases, respectively. Due to clinical relevance of these parameters, we performed an additional multivariable analysis, including ER/Her2 status. This analysis confirmed that prognostic relevance of *CASP8* -652 6N InsDel polymorphism is independent from ER or Her2 status (Additional file 1).

In conclusion, we revealed the *CASP8* -652 deletion variant to be an allele-dose dependent negative prognostic factor for patients with breast cancer. Moreover, homozygosity for the -652 6N del variant is an independent predictor for decreased OS.

### Prognostic relevance of *CASP8* Asp302His for breast cancer

In the following, we analyzed prognostic relevance of the *CASP8* Asp302His polymorphism in our study cohort. We observed that 151/200 patients (75.5 %) had an AspAsp genotype, 46/200 patients (23 %) were heterozygous (AspHis) and 3/200 patients (1.5 %) exhibited the rare HisHis genotype (Table 3). No deviation from Hardy-Weinberg equilibrium was detectable (*p* = 0.812) and the observed genotype distribution as well as the allelic frequencies (f_His_ = 0.130) were comparable to those previously reported in cancer cases and healthy controls of European ancestry [6, 7]. After confirming that clinico-pathological characteristics consistently lacked significant associations with the underlying genotypes (Table 3), Kaplan-Meier analysis was performed, in order to determine prognostic relevance of the *CASP8* Asp302His polymorphism. Interestingly, an allele-dose dependent influence of *CASP8* Asp302His upon OS was observed (Fig. 1d; *p* = 0.015), with homozygous minor allele carriers at highest risk of death (hazard ratio (HR) = 4.746, 95 % confidence interval (CI) = 1.14–19.71 *p* = 0.032; Table 4). Moreover, multivariable Cox-regression analysis revealed the His/His genotype to be an independent prognostic factor for reduced OS (HR = 4.889, 95%CI = 1.10–21.76; *p* = 0.037; Table 4). Here again, by performing an additional multivariable analysis, including available ER and Her2 data, we could confirm that prognostic relevance of *CASP8* Asp302His polymorphism is independent from ER or Her2 receptor status (Additional file 1).

**Table 3:** Clinico-pathological characteristics at primary diagnosis and Asp302His genotype distribution

	All	*CASP8* Asp302His genotype			*P*-value
		Asp/Asp	Asp/His	His/His	
n (%)	200	151 (75.5)	46 (23.0)	3 (1.5)	
Age at diagnosis (years ± SD)	56.46 ± 12.1	57.46 ± 12.0	52.87 ± 11.7	61.33 ± 11.0	0.089
Tumor type					
Ductal	136 (68.0)	109 (80.1)	24 (17.6)	3 (2.2)	0.086
Lobular	43 (21.5)	29 (67.4)	14 (32.6)	0 (0)	
Others	21 (10.5)	13 (61.9)	8 (38.1)	0 (0)	
Tumor size (mm ± SD)	24.20 ± 16.8	23.54 ± 17.4	25.22 ± 14.4	50.00 ± 7.1	0.163
Tumor stage					
pT_1_	107 (53.5)	84 (78.5)	22 (20.6)	1 (0.9)	0.321
pT_2_	71 (35.3)	49 (69.0)	21 (29.6)	1 (1.4)	
pT_3+4_	22 (11.0)	18 (81.8)	3 (13.6)	1 (6.7)	
Lymph node status					
pN_0_	115 (57.5)	92 (80.0)	22 (19.1)	1 (0.9)	0.202
pN_+_	85 (42.5)	59 (69.4)	24 (28.2)	2 (2.4)	
UICC stage					
I	76 (38.0)	59 (77.6)	16 (21.1)	1 (1.3)	0.387
II	78 (39.0)	58 (74.4)	20 (25.6)	0 (0)	
III + IV	46 (23.0)	34 (73.9)	10 (21.7)	2 (4.3)	
Grade					
1	71 (37.4)	56 (78.9)	15 (21.1)	0 (0)	0.459
2	68 (35.8)	49 (72.1)	18 (26.5)	1 (1.5)	
3	51 (26.8)	38 (74.5)	11 (21.6)	2 (3.9)	
Estrogen receptor status					
negative	53 (32.5)	43 (26.4)	10 (6.1)	0 (0)	
positive	110 (67.5)	81 (49.7)	27 (16.6)	2 (1.2)	0.402
Her2 status					
negative	138 (83.6)	104 (63.0)	32 (19.4)	2 (1.2)	
positive	27 (16.4)	21 (12.7)	6 (3.6)	0 (0)	0.811
Treatment					
Surgical treatment					
breast conserving	50 (25.0)	41 (82.0)	8 (16.0)	1 (2.0)	0.300
ablative	150 (75.0)	110 (73.3)	38 (25.3)	2 (1.3)	
Adjuvant therapy					
no adjuvant therapy	112 (56.0)	90 (80.4)	21 (18.8)	1 (0.9)	0.065
Tam and/or CMF	88 (44.0)	61 (69.3)	25 (28.4)	2 (2.3)	
Tam and/or CMF	88 (44.0)	61 (69.3)	25 (28.4)	2 (2.3)	

*Tam* Tamoxifen, *CMF* cyclophosphamide, methotrexate and 5 fluorouracil. Data are numbers with percentages given in brackets. Categorical variables were analyzed by χ^2^ statistics. *P* values were calculated using ANOVA for continuous variables

**Table 4:** Risk of death by uni- and multivariable Asp302His Cox-regression analyses

Variable	Hazard Ratio	95 % CI	*P*
*Univariate Analysis*			
Asp302His			
Asp/Asp	1^a^		
Asp/His	1.607	0.96–2.69	0.071
His/His	4.746	1.14–19.71	0.032
*Multivariable Analysis*			
Asp302His			
Asp/Asp	1^a^		
Asp/His	1.089	0.62–1.93	0.769
His/His	4.889	1.10–21.76	0.037
Age (per year)	1.002	0.98–1.02	0.839
Tumor type			
ductal	1^a^		
lobular	1.659	0.87–3.15	0.123
others	1.813	0.90–3.67	0.098
Tumor stage			
T_1_	1^a^		
T_2-4_	1.849	1.11–3.07	0.017
Nodal status			
negative	1^a^		
positive	3.652	2.13–6.27	<0.001
Grade			
1	1^a^		
2	1.020	0.55–1.91	0.952
3	1.648	0.82–3.30	0.158

^a^Reference group

In conclusion, we revealed the *CASP8* Asp302His variant to be an allele-dose dependent and negative prognostic factor for patients with breast cancer. Moreover, homozygosity for *CASP8* 302His variant is an independent predictor for decreased OS.

### Combined analysis of *CASP8* Asp302His and *CASP8* -652 6N InsDel and its prognostic relevance for breast cancer

We used Haploview to analyze putative linkage of the polymorphisms. We identified four different haplotypes, two common haplotypes (Del/Asp, f_Del/Asp_ = 0.448 and Ins/Asp, f_Ins/Asp_ = 0.422), the Del/His haplotype with a frequency of 0.115 and a rare haplotype (Ins/His f_Ins/His_ = 0.015). Since this analysis showed that *CASP8* Asp302His and *CASP8* -652 6N InsDel are in linkage disequilibrium to each other (D’ = 0.754), but showed a low correlation (*r*^*2*^ = 0.073), we inquired, whether the effects of these two polymorphisms are independent from each other. Interestingly, a Cox model including both polymorphisms revealed that homozygosity for *CASP8* -652 DelDel (HR = 2.384; 95%CI = 1.14–4.97; *p* = 0.020) and *CASP8* 302His (HR = 4.495, 95%CI = 1.07–18.94, *p* = 0.041) were both prognostic factors, which are independent from each other (Table 5).

**Table 5:** Risk of death by bivariate and combined multivariable Cox-regression analyses

Variable	Hazard Ratio	95 % CI	*P*
*Bivariate Analysis*			
-652 6N del			
InsIns	1^a^		
InsDel	1.178	0.57–2.44	0.660
DelDel	2.384	1.14–4.97	0.020
Asp302His			
Asp/Asp	1^a^		
Asp/His	1.439	0.85–2.44	0.175
His/His	4.495	1.07–18.94	0.041
*Multivariable Analysis*			
-652 + 302			
InsIns + AspAsp	1^a^		
InsDel + AspAsp	1.695	0.75–3.82	0.202
Ins-allele + His-allele	1.713	0.70–4.22	0.242
DelDel + AspAsp	3.129	1.39–7.05	0.006
DelDel + His-allele	2.961	1.17–7.53	0.023
Age (per year)	1.002	0.98–1.02	0.876
Tumor type			
ductal	1^a^		
lobular	1.933	1.01–3.71	0.048
others	1.714	0.85–3.45	0.130
Tumor stage			
T_1_	1^a^		
T_2-4_	1.794	1.08–2.98	0.024
Nodal status			
negative	1^a^		
positive	3.709	2.14–6.43	<0.001
Grade			
1	1^a^		
2	1.030	0.55–1.92	0.926
3	1.789	0.89–3.59	0.101

^a^Reference group

Moreover, to investigate prognostic significance of combined *CASP8* -652 6N InsDel and *CASP8* Asp302His genotypes in breast cancer patients, we used *CASP8* diplotypes (Fig. 1e). Theoretically, 4 haplotypes, as identified for these polymorphisms, lead to 10 diplotypes. However, due to the shown haplotype frequencies and the detected linkage of these polymorphisms, only 5 common diplotypes could be detected. Five patients belonged to rare diplotypes and needed to be analyzed together with other patients. Figure 1e shows how we joined these rare diplotype carriers with the common ones.

Kaplan-Meier analysis was performed, in order to determine prognostic relevance of *CASP8* diplotypes (Fig. 1f). We observed an additive influence of *CASP8* Asp302His and *CASP8* -652 6N InsDel upon OS (*p* = 0.0002). Consequently, individuals bearing a -652 DelDel and a homo- or heterozygous 302His diplotype had the highest risk of death, followed by patients with a -652 DelDel variant and 302 AspAsp diplotype.

Moreover, the presence of the -652 DelDel variant and a homo- or heterozygous 302His diplotype or the presence of the -652 6N del variant and the 302 AspAsp diplotype were independent predictors for OS (HR = 3.129, 95%CI = 1.39–7.05; *p* = 0.006; HR = 2.961, 95%CI = 1.17–7.53; *p* = 0.023, respectively, Table 5). Including available ER and Her2 data in an additional multivariable analysis confirmed that prognostic relevance of *CASP8* diplotypes is independent from ER or Her2 receptor status (Additional file 1).

Thus, we may conclude that the *CASP8* -652 6N Del or the *CASP8* Asp302His variant provide an allele-dose dependent and negative prognostic factor for breast cancer, independently from each other.

## Discussion

In the present study, we investigated clinical relevance of two selected caspase 8 polymorphisms, namely *CASP8* -652 6N InsDel and Asp302His, for patients with primary breast cancer. Intriguingly, in contrast to previous molecular epidemiological findings [4, 7, 26], describing an association of the *CASP8* -652 6N deletion variant or the *CASP8* Asp302His variant with decreased breast cancer susceptibility, we showed that these caspase 8 variants have a negative and allele-dose dependent prognostic impact on breast cancer overall survival. Moreover, we confirmed that clinical informativity of both polymorphisms is independent from each other and that these polymorphisms have, besides, an allele-dose dependent additive influence on OS.

Considering that activation induced cell-death of antitumor T-lymphocytes was shown to be involved into immune surveillance of cancer cells [3, 27], the functionally underlying death receptor-pathway emerged as an interesting target to seek novel candidate polymorphisms for cancer susceptibility. In this regard, *CASP8* -652 6N InsDel has already been shown to have an influence on caspase 8 mRNA expression in stimulated T-lymphocytes, by disrupting a Specifity Protein 1 (Sp1) transcription factor binding site in the caspase 8 promoter region and, consequently, by functionally interfering with caspase 8 transcription [3]. Complementarily, we reported that breast cancer tissues of patients, bearing a homozygous -652 6N Del variant, displayed lowest relative *CASP8* expression, which corroborates that this effect is similarly applicable for malignant breast cancer tissue. This finding was not necessarily anticipated. Although Sp1 sites are typically believed to represent constitutive promoter elements for basal transcription, recent studies showed that, especially in cancer, the Sp1 transcription factor can be strongly regulated by post-translational modifications that positively or negatively affect its activity on a wide array of genes [28, 29].

The *CASP8* Asp302His variant, especially in form the His/His genotype, was a rare event in our study population, which is in accordance to previous independent observations [5]. Similarly, *CASP8* 302His variant was shown to confer reduced breast cancer susceptibility in an allele-dose dependent manner [4]. However, given that the functional effect of this polymorphism is largely unknown, the underlying effect on caspase 8 functionality and tumor progression is less clear. Nevertheless, aspartate 302 was shown to be conserved between mouse and human caspase 8 and is located on the protein surface. Therefore, it has already been hypothesized that the Asp302His change could likewise impair caspase 8 function, possibly by negatively affecting its auto processing capability or its catalytic activity [5]. However, albeit highly interesting, a detailed functional analysis of the polymorphisms, investigated herein, is beyond the objective of our present investigation.

As our key finding, we described both caspase 8 variants as a negative prognostic factor for breast cancer. At first glance, our finding may appear counterintuitive, since the *CASP8* -652 InsDel or DelDel genotype has previously been associated with impaired immune surveillance of cancer cells and concomitantly decreased breast cancer susceptibility [3, 6, 7, 9]. However, our data are in accordance with a recent pilot investigation, reporting, albeit with borderline statistical significance, a negative prognostic impact of the *CASP8* -652 6N Del allele for colorectal cancer patients [19]. Complementarily, in a very recent approach, *CASP8* 302His was associated with worse overall and event-free survival in patients with MYCN-amplified neuroblastoma tumors [18]. Apoptosis, with caspase 8 as one of its key regulators, is not only involved in AICD of antitumor T lymphocytes, but also constitutes an important defense mechanism against hyperproliferation and malignancy, which can be induced by e.g. DNA damage [30, 31]. Therefore, the acquired ability to resist apoptotic stimuli, caused by aberrations in key apoptotic pathways, is an essential characteristic for cells to become malignant and to develop a metastatic phenotype [31, 32]. Moreover, the death receptor pathway, with caspase 8 as key regulator, was shown to be de-regulated in malignant tumor cells, such as in breast cancer cells [33, 34], in dysplastic cells or in carcinomas in situ [35]. Therefore, in breast cancer, we hypothesize an ambiguous tumor biological relevance and a context dependent clinical informativity for *CASP8* -652 InsDel: In healthy individuals, impaired caspase 8 activity and reduced apoptotic capacity seems to have primarily influence on immune escape (in terms of AICD) and obviously decreases breast cancer susceptibility. Contrarily, in patients with diagnosis of primary breast cancer, in which malignant cells have already accomplished immune escape, the so far protective effect of increased immune surveillance becomes obviously inferior. In this situation, increased resistance of tumor cells to apoptotic stimuli, conferred by the *CASP8* InsDel or DelDel genotype, turns the balance and becomes a potentially pro-tumorigenic and negative prognostic factor, resulting in decreased OS. However, this concept is not necessarily transferable to other cancer entities, since the *CASP8* InsDel and DelDel genotypes were contrarily described as favorable prognostic indicators for gastric cancer patients [36]. However, in the light of the complexity of death receptor signaling, these data are not surprising. It is known that the magnitude of pro-death events (such as caspase activation) and pro-survival events (such as Nuclear Factor (NF)-kB) may vary not only from one cell type to the next but also among individual cells of the same type due to intrinsic and extrinsic factors. Therefore, death receptor ligands may simultaneously activate opposing signals via the same receptors [37].

Moreover, albeit being in linkage disequilibrium, *CASP8* -652 6N InsDel and *CASP8* Asp302His showed an independent and additive prognostic impact on OS. Therefore, we may hypothesize that both polymorphisms may account for an additive or even synergistic effect on total caspase 8 activity in breast cancer cells.

## Conclusion

To the best of our knowledge, this is the first report describing a prognostic impact of both *CASP8* -652 6N InsDel and *CASP8* Asp302His for breast cancer patients. However, considering the limited number of patients in our study, our statistically verified conclusions should be handled with care and our explorative approach needs to be clinically validated in larger and independent patient cohorts. Nevertheless, we performed multivariate analysis to reduce the risk of accidental findings, which revealed that our results remained significant after correction for the covariates. Moreover, two completely independent outcome studies in neuroblastoma and colon cancer showed a comparable effect of -652 6N InsDel and Asp302His, respectively [18, 19]. This provides further evidence for a real functional effect of these polymorphisms. Therefore, further larger (prospective) studies should be initiated to validate clinical utility of these two *CASP8* polymorphisms for breast cancer and also to a broad scope of other malignancies.

## Abbreviations

AICD, activation induced cell death; CI, confidence intervall; DISC, death inducing signalling complex; HR, hazard ratio; NF, nuclear factor; OS, overall survival; PCR, polymerase chain reaction; Sp1, specifity protein 1

## Additional files

Additional file 1: Multivariate analysis including Estrogen and Her2 receptor status. This additional file comprises 3 sub-tables in total. In Table 1, risk of death is calculated by multivariate -652 6N del Cox-regression analyses including Estrogen receptor and Her2 receptor status. In Table 2, risk of death is calculated by multivariate Asp302His Cox-regression analyses including Estrogen receptor and Her2 receptor status. In Table 3, risk of death is calculated by *CASP8* diplotypes multivariate Cox-regression including Estrogen receptor and Her2 receptor status. (DOCX 22 kb) Additional file 2: Anonymized clinical dataset. All variables used for haplotype and diplotype construction, Kaplan-Meier curves, log-rank tests, univariate and multivariable analyses. Ages were replaced by age ranges to maintain participant confidentiality. (XLSX 20 kb)
