# Supplementary material for: Prognostic relevance of caspase 8 -652 6N InsDel and Asp302His polymorphisms for breast cancer
Source: BMC Cancer. 2016 Aug 9;16:618. doi: 10.1186/s12885-016-2662-x (PMC4977759; doi:10.1186/s12885-016-2662-x)
Supplement: Additional file 1: — Multivariate analysis including Estrogen and Her2 receptor status. This additional file comprises 3 sub-tables in total. In Table 1, risk of death is calculated by multivariate -652 6N del Cox-regression analyses including Estrogen receptor and Her2 receptor status. In Table 2, risk of death is calculated by multivariate Asp302His Cox-regression analyses including Estrogen receptor and Her2 receptor status. In Table 3, risk of death is calculated by CASP8 diplotypes multivariate Cox-regression including Estrogen receptor and Her2 receptor status. (DOCX 22 kb) [file 12885_2016_2662_MOESM1_ESM.docx]

| **Variable** | **Hazard Ratio** | **95% CI** | ***P*** |
| --- | --- | --- | --- |
| -652 6N del |  |  |  |
| Ins/Ins | 1* |  |  |
| Ins/Del | 1.547 | 0.68-3.53 | 0.300 |
| Del/Del | 2.635 | 1.13-6.13 | 0.025 |
| Age (per year) | 1.012 | 0.99-1.04 | 0.358 |
| Tumor type |  |  |  |
| ductal | 1* |  |  |
| lobular | 1.841 | 0.87-3.92 | 0.113 |
| others | 2.800 | 1.33-5.90 | 0.007 |
| Tumor stage |  |  |  |
| T_1_ | 1* |  |  |
| T_2-4_ | 2.312 | 1.30-4.12 | 0.004 |
| Nodal status |  |  |  |
| negative | 1* |  |  |
| positive | 3.688 | 2.04-6.68 | <0.001 |
| Grade |  |  |  |
| 1 | 1* |  |  |
| 2 | 0.875 | 0.44-1.75 | 0.705 |
| 3 | 1.233 | 0.57-2.69 | 0.599 |
| Estrogen receptor status |  |  |  |
| negative | 1* |  |  |
| positive | 0.310 | 0.16-0.59 | <0.001 |
| Her2 status |  |  |  |
| negative | 1* |  |  |
| positive | 0.539 | 0.24-1.19 | 0.125 |
|  | | | |
| *Reference group. | | | |

**Supplementary Table 1: Risk of death by multivariate -652 6N del Cox-regression analyses including Estrogen receptor and Her2 receptor status**

| **Variable** | **Hazard Ratio** | **95% CI** | ***P*** |
| --- | --- | --- | --- |
|  |  |  |  |
| *Multivariate Analysis* |  |  |  |
| Asp302His |  |  |  |
| Asp/Asp | 1* |  |  |
| Asp/His | 1.171 | 0.62-2.23 | 0.631 |
| His/His | 14.108 | 2.63-75.73 | 0.002 |
| Age (per year) | 1.011 | 0.99-1.04 | 0.386 |
| Tumor type |  |  |  |
| ductal | 1* |  |  |
| lobular | 1.799 | 0.85-3.82 | 0.127 |
| others | 3.445 | 1.57-7.58 | 0.002 |
| Tumor stage |  |  |  |
| T_1_ | 1* |  |  |
| T_2-4_ | 2.216 | 1.24-3.96 | 0.007 |
| Nodal status |  |  |  |
| negative | 1* |  |  |
| positive | 3.536 | 1.95-6.40 | <0.001 |
| Grade |  |  |  |
| 1 | 1* |  |  |
| 2 | 0.960 | 0.48-1.94 | 0.910 |
| 3 | 1.110 | 0.50-2.47 | 0.798 |
| Estrogen receptor status |  |  |  |
| negative | 1* |  |  |
| positive | 0.299 | 0.16-0.57 | <0.001 |
| Her2 status |  |  |  |
| negative | 1* |  |  |
| positive | 0.620 | 0.28-1.38 | 0.240 |
|  | | | |
| *Reference group. | | | |

**Supplementary Table 2: Risk of death by multivariate Asp302His Cox-regression analyses including Estrogen receptor and Her2 receptor status**

| **Variable** | | **Hazard Ratio** | **95% CI** | ***P*** |  |
| --- | --- | --- | --- | --- | --- |
|  | |  |  |  |  |
| *Multivariate Analysis* | |  |  |  |  |
| -652 + 302 | |  |  |  |  |
| Ins/Ins + Asp/Asp | | 1* |  |  |  |
| Ins/Del + Asp/Asp | | 1.765 | 0.71-4.37 | 0.219 |  |
| Ins-allele + His-allele | | 1.942 | 0.69-5.44 | 0.207 |  |
| Del/Del + Asp/Asp | | 2.964 | 1.16-7.61 | 0.024 |  |
| Del/Del + His-allele | | 3.106 | 1.09-8.86 | 0.034 |  |
| Age (per year) | | 1.012 | 0.99-1.04 | 0.362 |  |
| Tumor type | |  |  |  |  |
| ductal | | 1* |  |  |  |
| lobular | | 1.849 | 0.87-3.95 | 0.112 |  |
| others | | 2.694 | 1.25-5.81 | 0.011 |  |
| Tumor stage |  | |  |  |  |
| T_1_ | 1* | |  |  |  |
| T_2-4_ | 2.290 | | 1.28-4.09 | 0.005 |  |
| Nodal status | |  |  |  |  |
| negative | | 1* |  |  |  |
| positive | | 3.690 | 2.02-6.76 | <0.001 |  |
| Grade | |  |  |  |  |
| 1 | | 1* |  |  |  |
| 2 | | 0.845 | 0.42-1.71 | 0.639 |  |
| 3 | | 1.204 | 0.55-2.65 | 0.645 |  |
| Estrogen receptor status | |  |  |  |  |
| negative | | 1* |  |  |  |
| positive | | 0.308 | 0.16-0.59 | <0.001 |  |
| Her2 status | |  |  |  |  |
| negative | | 1* |  |  |  |
| positive | | 0.551 | 0.25-1.24 | 0.149 |  |
| *Reference group. | | | | |  |

**Supplementary Table 3: Risk of death by combined multivariate Cox-regression including Estrogen receptor and Her2 receptor status**
